# Susceptibility to Enterocins and Lantibiotic Bacteriocins of Biofilm-Forming Enterococci Isolated from Slovak Fermented Meat Products Available on the Market

**DOI:** 10.3390/ijerph17249586

**Published:** 2020-12-21

**Authors:** Andrea Lauková, Anna Kandričáková, Eva Bino

**Affiliations:** Institute of Animal Physiology, Centre of Biosciences of the Slovak Academy of Sciences, Šoltésovej 4-6, 040 01 Košice, Slovakia; anicka13@gmail.com (A.K.); glatzova@saske.sk (E.B.)

**Keywords:** dry fermented meat products, enterococci, bacteriocins, susceptibility, biofilm

## Abstract

This study investigated eight types of Slovak dry fermented meat products (salami and sausages) that are available on the market and were produced by three different producers in different regions of Slovakia. The total counts of enterococci in these products ranged from 2.0 up to 6.0 cfu/g (log10). Three species were identified among the 15 selected enterococcal strains; *Enterococcus faecium* (8 strains), *Enterococcus faecalis* (3) and *Enterococcus hirae* (4). They were hemolysis-negative (γ-hemolysis) with a biofilm-forming ability, which was evaluated as low-grade biofilm formation, susceptible to conventional antibiotics and mainly susceptible to lantibiotic bacteriocins, namely, gallidermin and nisin; they even showed a higher susceptibility to gallidermin than to nisin. They were also susceptible to enterocin–durancin, but most strains showed resistance to enterocin A/P. This study indicated that bacteriocins can play a key role in preventing and/or protecting from undesirable bacterial multiplication or contamination in the food industry and that they have great potential for further experimental applications.

## 1. Introduction

Sausages and salami are dry fermented meat products that are very popular and widely consumed among the populations of many countries. In this field, the production of especially traditional fermented meat products is mostly associated with the Mediterranean countries [1]. However, in Slovakia, sausages and salami are also popular among consumers. Many different types of these products are available on the market. The microbiota in these meat products is diverse and is influenced by the natural microorganisms present in the raw material, the pH, temperature, water activity and the fermentation process itself [2]. The autochthonous microbiota is dominated by lactic acid bacteria (LAB), with the presence of enterococci being mainly attributed to their phenotypic features (proteolytic/lipolytic activities, carbohydrate metabolism, low acidification ability), which may lead to an improvement in the fermented products’ characteristics [3,4]. On the one hand, enterococci can have beneficial/probiotic properties with their production of bacteriocins [5,6,7,8,9]; on the other hand, some strains can carry virulence factors and they can contribute to the spoilage of products under certain conditions [4]. So far, 59 enterococcal species have been validated; Tanasupawet et al. [10] and Franz et al. [5] reported an enterococcal species group based on 16S rRNA gene similarity, although up to now, not all validated species have been grouped this way. Enterococci from the genus *Enterococcus* belong in the family Enterococcacae, order Lactobacillales, class Bacilli and phylum Firmicutes. They are also involved in the lactic acid bacteria group because of their lactic acid production [5]. On the one hand, enterococci can withstand extreme conditions by adapting various matrices; however, under some conditions, they can cause spoilage of those matrices. From this point of view, knowing the possibilities for their prevention/reduction is often crucial. Knowing the inhibition spectra and non-residual character of bacteriocins, it is interesting to find out whether enterococci from various sources with different properties can be susceptible to bacteriocins as an alternative means of prevention/protection; for example, by treating/eliminating contaminant bacteria in food. Previously, some *in vitro* studies, as well as *in vivo*/*in situ* studies, have been conducted on enterococci, which confirmed their anti-contaminant bacteria effectivity [9,11,12,13]. In this study, therefore, enterococci isolated from different Slovak dry fermented meat products produced in different workplaces that are available on the market were investigated. The main aim of this study was to evaluate those enterococci for their susceptibility to enterocins and lantibiotic bacteriocins; at first, however, isolates were taxonomically allotted and assessed regarding their hemolysis, enzyme production, biofilm formation abilities and antibiotic profile.

## 2. Materials and Methods

### 2.1. Count of the Enterococci and Their Identification

Eight different types of Slovak dry fermented meat products made by three different producers that were available on the market were used in this study, namely, the salami types known as Bratislavska, Nitran, Kalinka, Plesnivec, Scipak, Tokaj and Lovecka, as well as Kľusovska sausage. Their general/brief description is as follows: Bratislavska salami is made with a 96% mixture of meats (pork and beef), salt, antioxidants and a starter culture. Plesnivec salami is also produced with a starter culture, prepared from a mixture of meats (mostly pork), additives, dextrose and spices. Similarly, Kalinka salami contains 45% pork, 23% beef, 22% bacon/fat, garlic, spice, salt and a starter culture. The next products are produced without a starter culture: Scipak belongs among the products containing 51% pork, 27% beef, 17% bacon/fat, salt and pepper; Nitran salami is also made from a pork and beef mixture (96%), salt, spice mixture, garlic, a stabiliser and an antioxidant (ascorbic acid). Tokaj salami contains pork and spices. Lovecka salami with a smoked flavor contains pork and spices and smoked Kľusovska sausage is made from pork, bacon/fat, spices and dried vegetables. The products were bought on the market and taken to our laboratory. They were sampled and treated using the standard microbiological dilution method following the International Organization for Standardization (ISO) protocol; samples (10 g) were mixed in 90 mL of peptone water (ISO 6579, Merck, Darmstadt, Germany), treated using a Stomacher (IUL, Masticator, Spain) and diluted (1:9). Dilutions were spread onto M-Enterococcus agar plates (ISO-7889, Difco, Sparks, MD, USA) and cultivated at 37 °C for 48 h in a 5% gaseous atmosphere (CO_2_/air). The total count of enterococci was expressed in colony-forming units per millilitre (cfu/mL). Representative colonies were picked up, inoculated on Brain-Heart-Blood Agar (Difco, Sparks, MD, USA) to check their purity. Pure colonies were submitted for identification using a matrix–assisted laser desorption ionisation time-of-flight spectrometry identification system (MALDI-TOF MS, Brucker Daltonics, Billerica, MD, USA) based on protein “fingerprints” [14]. Lysates of bacterial cells were prepared according to the producer’s instructions (Bruker Daltonics). The results were evaluated using the MALDI Biotyper 3.0 identification database (Bruker Daltonics). According to their score values, the identified strains were taxonomically allocated on the basis of highly-probable species identification (score 2.300–3.000), secure genus identification/probable species identification (2.000–2.299) and probable genus identification (1.700–1.999). Positive controls were those included in the Bruker Daltonics database. Identical colonies evaluated with the same MALDI-TOF score values were excluded.

Besides the MALDI-TOF identification, the polymerase chain reaction (PCR) genotyping method (Techgene, United Kingdom) was applied, followed by agarose electrophoresis in 0.8% agarose gel (Sigma-Aldrich, Darmstadt, Germany) buffered with 1×TAE (Tris Acetate EDTA buffer, Merck) containing 1 µg/mL ethidium bromide (Sigma-Aldrich). The molecular mass standard (Promega, Madison, WI, USA) was used according to the manufacturer’s instructions. A DNA template from each strain was isolated by means of rapid alkaline lysis [15]. The sequences of the primer pairs used for PCR amplification of *E. hirae* were 5′-TCTTGATGCCGATG-3′ and 5′-ATCCTTCGCGGAAT-3′ [16]; for *E. faecium*, 5′-GCAAGCTTCTTAGAGA-3′ and 5′-CATCGTGTAAGCTAACTTC-3′ (Invitrogen, MS, USA) were used; for *E. faecalis,* 5′-ATCAAGTACAGTTAGTCTT-3′ and 5′-ACGATTCAAAGCTAACTG-3′ were used, following the protocol according to Woodford et al. [17]. The positive controls were *E. hirae* DCH5 [18] and our strain CCM 7419 of *E. faecium* EK13, as well as *E. faecalis* CCM 4224 (Czech Culture Collection, Brno, Czech Republic). The identified strains were maintained on M-Enterococcus agar (Difco, Sparks, MD, USA). Strains were stored using the Microbank system (Pro-Lab Diagnostic, Richmond, BC, Canada) and used for subsequent analyses.

### 2.2. Enzyme Production and Hemolysis

The commercial API-ZYM system (BioMérioux, Marcy l’Etoile, France) was used to test the enzyme production. According to the manufacturer‘s recommendation, the following enzymes were tested: alkaline phosphatase, esterase (C4), esterase lipase (C8), lipase (C14), leucine arylamidase, valine arylamidase, cystine arylamidase, trypsin, α-chymotrypsin, acid phosphatase, naftol-AS-BI-phosphohydrolase, α-galactosidase, β-galactosidase, β-glucuronidase, α-glucosidase, β-glucosidase, *N*-acetyl-β-glucosamonidase, α-manosidase and α-fucosidase. Inocula (65 µL) of McFarland Standard 1 suspensions were pipetted into each well of the kit. Enzyme activities were evaluated after 4 h of incubation at 37 °C and after the addition of Zym A and Zym B reagents. Color intensity values from 0 to 5 and their relevant value in nanomoles were assigned for each reaction according to the color chart supplied with the kit.

Hemolysis was detected by streaking the cultures onto BH agar (Difco, USA) and supplemented with 5% defibrinated sheep blood. Plates were incubated at 37 °C for 24 h in an incubator. The presence or absence of clear zones around the colonies was interpreted as α- and β-hemolysis respectively, while γ-hemolysis indicated negative strains [19].

### 2.3. Biofilm Formation Testing

A quantitative plate assay [20,21] was used to test the biofilm formation ability in the enterococci. One colony of each strain grown on Brain-Heart-Blood Agar overnight at 37 °C (Difco, Sparks, MD, USA) was transferred into 5 mL of Ringer solution (pH 7.0, 0.75% *w*/*v*) to obtain a suspension corresponding to 1 × 10^8^ cfu/mL. A 100 µL aliquot from that dilution was transferred into 10 mL of Brain Heart Infusion/Broth (BHI, Difco, Sparks, MD, USA). A 200 µL volume of the dilution was inoculated into polystyrene microtiter plate wells (Greiner ELISA 12 Well Strips, 350 µL, flat bottom, Frickenhausen GmbH, Frickenhausen, Germany) and incubated for 24 h at 37 °C. The biofilm that formed in the microtiter plate wells was washed twice with 200 µL of deionized water and dried at 25 °C for 40 min. The remaining attached bacteria were stained for 30 min at 25 °C with 200 µL 0.1% (*m*/*v*) crystal violet in deionized water. The dye solution was aspirated away, and the wells were washed twice with 200 µL of deionized water. After the water removal, the plate was dried for 30 min at 25 °C and the dye bound to the adherent biofilm was extracted with 200 µL 95% ethanol. A 150 µL aliquot was transferred from each well into a new microplate well for absorbance (A) at 570 nm using an Apollo 11 Absorbance Microplate reader LB 913 (Apollo, Berthold Technologies, Oak Ridge, TN, USA). Each strain and condition was tested in two independent tests with 12 replicates. Sterile BHI was included in each analysis as a negative control. *Streptococcus equi* subsp. *zooepidemicus* CCM 7316 was used as a positive control (kindly provided by Eva Styková, University of Veterinary Medicine and Pharmacy, Košice, Slovakia). Biofilm formation was classified as highly positive (A_570_ ≥ 1.0), low-grade positive (0.1 ≤ A_570_ < 1.0) or negative (A_570_ < 0.1) according to Chaieb et al. [20] and Slížová et al. [21].

### 2.4. Antibiotic Profile

Following the European Food Safety Authority (EFSA) rules for determining antibiotic profiles, the Clinical Laboratory Standard Institute (CLSI) [22] system was applied with antibiotic disks. Antibiotics (13) (Oxoid, Basingstoke, Hampshire, United Kingdom) were used according to the supplier’s recommendations as the basic targeting antibiotics used in clinical testing: clindamycin (2 µg), novobiocin (Nov, 5 µg), ampicillin (AMP, 10 µg), penicillin (10 IU), erythromycin, azithromycin (E, Azm, 15 µg), streptomycin (S, 25 µg), chloramphenicol, rifampicin, vancomycin, tetracycline, kanamycin (C, RIF, VAN, T, KAN, 30 µg) and gentamicin (Gn, 120 µg). An overnight culture (100 µL) of strains were spread on Mueller-Hinton agar enriched with 5% of defibrinated sheep blood (Difco, Sparks, MD, USA). The disks were placed on agar plates and cultivated at 37 °C for 18 h. The susceptibility to antibiotics was read by measuring the inhibition zones in mm and evaluated according to the disk supplier and the EFSA table. The positive strains were *E. faecium* CCM 7419 [23], *E. faecalis* CCM 4224 (from CCM, Brno, Czech Republic) and *E. hirae* DCH5 [18].

### 2.5. Susceptibility to Enterocins and Lantibiotic Bacteriocins

Ent A/P and durancin ED26E/7 are bacteriocins classified in group II [6]; they are thermostable enterocins with a broad antimicrobial spectrum. To treat the indicator strains, we used Ent A/P that was prepared according to Mareková et al. [23]. It is produced by *E. faecium* EK13 (CCM 7419). Durancin ED26E/7 was prepared as previously described by Lauková et al. [24]. It is produced by *Enterococcus durans* [24]. Gallidermin (a pure substance supplied by Enzo Life Sci. Corporation, Farmingdale, NY, USA, MW2069.4) and Nisin (Nisaplin, Aplin and Barret, Trowbridge, United Kingdom) were used as previously reported by Lauková et al. [25]. Because of the purity of the gallidermin (evaluated from previous tests), it was used at a concentration of 0.5 mg/mL in 2 µL doses. Similarly, Nisaplin contains nisin with an activity of 1,000,000 IU; however, it was applied at a concentration of 1 mg/mL and in 10 µg doses. Enterocins were used at the same concentration and dosage as nisin. The susceptibility to enterocins and lantibiotic bacteriocins of the identified enterococci was tested using the agar spot diffusion method [26]. Briefly, Brain-Heart Infusion/Broth supplemented with 1.5% agar (BHIA, Difco, Sparks, MD, USA) was used for the bottom layer. For the overlay, 0.7% BHIA was used enriched with 200 µL of an 18 h culture of an indicator strain (to have an A_600_ absorbance up to 0.800 nm). A dilution of bacteriocins in phosphate buffer (pH 6.5, ratio 1:1) was dropped on the soft agar surface with each tested enterococcal indicator strain. The plates were incubated at 37 °C for 18 h. Clear inhibition zones around the doses of bacteriocins were checked and the inhibition activity was expressed in arbitrary units per milliliter (AU/mL); this means the reciprocal of the highest two-fold dilution of bacteriocins demonstrating complete growth inhibition of the indicator strain. Tests were performed twice. The positive control was the principal indicator strain *Enterococcus avium* EA5 (our isolate from piglets); its growth was inhibited by an activity of 25,600 AU/mL.

## 3. Results

Total enterococcal count in Kalinka salami reached 2.0 cfu/g (log10), while in Scipak and Tokaj salami, it was 3.3 and 3.0 cfu/g, respectively; in Kľusovska sausage, it was determined to be 3.48 cfu/g. In Bratislavska and Nitran salami, we counted enterococci in volumes of 4.8 and 4.4 cfu/g (log10), respectively. Lovecka and Plesnivec revealed high enterococcal amounts (5.2 and 6.08 cfu/g, respectively); however, this could have been due to the starter cultures used.

Six out of the 15 strains were taxonomically allotted on the basis of highly probable species identification (2.300–3.000) and nine strains were assessed and allotted on the basis of secure genus identification/probable species identification (2.000–2.299). They were allotted to three species: *E. faecium* (8 strains), *E. faecalis* (3 strains) and *E. hirae* (4 strains, Table 1). In addition, PCR genotyping was performed and confirmed the species allocation for each strain, reaching 550 bp for *E. faecium*, 441 bp for *E. faecalis* and 463 bp for *E. hirae.* The *E. faecium* strains were isolated from the salami types Bratislavska (EF1BS), Nitran (EF1NS), Scipak (EF2SC) and Kalinka (EF2Kal, EF4Kal), as well as Kľusovska sausage (EFKL5) and Plesnivec salami (EFPL3, EFPL4). However, in Plesnivec salami, we also identified *E. faecalis* (EEPL1S) and *E. hirae* (EHPL2) strains. *E. faecalis* was also found in Kľusovska sausage (EEKL2) and Scipak salami (EE1Sc). *E. hirae* strain species were isolated from Tokaj salami (EHTOK1, EHTOK2) and Plesnivec salami (EHPL2, Table 1). *E. hirae* was also detected in Lovecka salami. In each type of fermented meat product involved in the testing, we identified an *Enterococcus* species; however, in Tokaj salami and Lovecka salami, only the species *E. hirae* was detected; in Bratislavska, Kalinka and Nitran salami, only the species *E. faecium* was found. In Plesnivec salami, three species were detected; in Kl′usovska sausage and Scipak salami, representatives of the species *E. faecium* and *E. faecalis* were found.

The strains were hemolysis negative (γ-hemolysis). They were susceptible to vancomycin, novobiocin, chloramphenicol, erythromycin and penicillin. They were resistant to kanamycin, rifampicin and streptomycin. Thirteen out of 15 strains were also resistant to Azm, except EHLov1 and EHTOK2, which were susceptible to Azm (inhibition zone sizes of 12 and 19 mm, respectively; Table 1). Similarly, the strains were susceptible to tetracycline (inhibition zone size ranged from 10 up to 20 mm), except *E. hirae* EHPL2, which was resistant to tetracycline. While some strains were resistant to clindamycine (Da), namely three *E. hirae* strains (EHLov1, EHPL2 and EHTOK2; Table 1) and one *E. faecium* strain (EF2Kal), the other strains were susceptible to Da, and except for two *E. faecalis* strains (EEPL1S, EE1Sc) that were resistant to gentamicin, the rest were susceptible to Gn. One *E. hirae* (EHTOK 2), one *E. faecalis* EEKL2) and two *E. faecium* (EF1NS, EFKL5) were resistant to Amp. EHPL2 showed resistance to three antibiotics, namely, Azm, T and Da, while the remaining strains were resistant to at least one antibiotic (Table 1).

Enterococci from dry fermented meat products were low in enzyme production. Regarding trypsin and α-chymotrypsin, almost no production was found, except for two strains (Table 2). The same situation appeared in the case of β-glucuronidase (5 nmoL was evaluated in EFPL4, EEKL2 and EHTOK1). The production of β-glucosidase was measured in all strains, although at a low level (5 nmoL) (Table 2). Esterase and esterase lipase were higher (20 nmoL in four strains of different species (EF1BS, EF2SC, EF2Kal and EE1Sc; Table 2). In general, these strains appear safe from the point of view of enzyme evaluation.

Four strains out of the 14 tested (one strain not tested) representing three species (*E. faecium*, *E. faecalis* and *E. hirae*) did not form a biofilm (Table 3). Ten strains (Table 3) were evaluated as being low-grade (0.1 ≤ A_570_ < 1.0) biofilm-forming strains (Table 3) with values from 0.113 up to 0.367. The highest biofilm ability was demonstrated in *E. faecalis* EE1Sc from Scipak salami (0.367 ± 0.08); the lowest value was measured in *E. faecium* strain EF1NS isolated from Nitran salami (0.113 ± 0.30).

Despite their resistance to some antibiotics, all strains were susceptible to gallidermin and nisin (Table 3). The inhibition activity evaluated in the case of gallidermin ranged from 200 AU/mL (in strain *E. faecalis* EE1Sc with the highest biofilm formation ability) up to 25,600 AU/mL (in strains *E. faecium* EF2Kal (biofilm not tested) and EF4Kal (biofilm not formed)). The range of nisin activity against enterococci varied from 800 AU/mL (in EFPL1S strain isolated from Plesnivec salami) up to 6400 AU/mL (Table 3). Judging from the inhibition activity values, enterococci showed higher susceptibility to gallidermin than to nisin.

Regarding the enterocins, most strains were resistant to Ent A/P produced by *E. faecium* EK13 (CCM7419; Table 3); the growth of only four strains (EFPL1S, EFPL3, EEKL2 and EHTOK1) was inhibited using Ent A/P with activity 100 AU/mL (Table 3). On the other hand, when using durancin ED26E/7 produced by *E. durans* (from ewe milk lump cheese), the growth of 14 strains was inhibited with activity ranging from 400 up to 6400 AU/mL (Table 3). *E. faecium* EFPL1S, EFPL3, *E. faecalis* EEKL2 and *E. hirae* EH TOK1 were susceptible to enterocins and lantibiotic bacteriocins.

## 4. Discussion

The presence of enterococci in the gastrointestinal tract of animals [7,27] leads to a high potential for meat contamination at the time of slaughtering [5,28]. However, enterococci not only contaminate raw meats but they can also be active in processed meat, including dry fermented meat products [3]. Microbiota in these meat products can be influenced by maturing; the enterococcal counts in the products studied varied from 2.0 to 6.08 cfu/g. Lauková et al. [29] reported on a different type of dry fermented salami from those investigated in this study, namely, the type called Start, with a lactic acid bacteria count of 6.25 cfu/g (log10). The species *E. faecalis* dominated the enterococcal species in chourico, a traditional sausage produced in southern Portugal [11]. Enterococci can withstand extreme temperatures and high salinity, and they tolerate bile salts or low pH [30]; this means they can adapt to various matrices; however, under some conditions, they can cause spoilage of those matrices, including foodstuffs. Three species detected, namely, *E. faecium*, *E. faecalis* and *E. hirae*, belong in two clusters based on the 16S rRNA method [5].

With regard to enzymes as disease markers, enterococci from dry fermented meat products appear not to cause spoilage because they produced no trypsin (except the *E. faecalis* strain EEKL 2–5 nmoL), with α-chymotrypsin (EEKL 2–5 nmoL) and especially no endo-β-*N*-acetylglucosaminidase. The latter enzyme is one that needs to proliferate in vivo. This enzyme cleaves mannose-type glycans in glycoproteins between the *N*-acetylglucosamine residues of the pentasaccharide core [31]. The enzyme β-glucuronidase was not produced by the tested enterococci, except by strains EFPL4, EEKL2 and EHTOK1 (5 nmoL). On the other hand, the beneficial enzyme β-galactosidase (lactase) was produced by all strains except EHPL2, albeit in a slight amount of 5 nmoL. This lactase is widely used in the dairy industry for the production of lactose-free milk for consumption by lactose-intolerant people. Enterococci typically exhibit γ-hemolysis (negative). Hemolysis-positive results are mostly associated with hemolysis gene presence, indicating virulence factor activity. Although gene presence was not checked here, γ-hemolysis indicates their most likely do not have a pathogenic character, which promotes the possibility for their testing for bacteriocin activity, for instance. Moreover, no γ-hemolysis was reported in enterococci isolated from raw goat milk [32].

Biofilms are sessile communities of bacteria that are typically embedded in an extracellular polymeric matrix [33]. The tested strains showed low-grade biofilm-forming ability or they did not form a biofilm at all. Low-grade or no biofilm-forming ability was also found in enterococci from raw goat milk [32]. However, the enterococci present there were susceptible to bacteriocin treatment, which is very promising with regard to the fight against biofilms. Mathur et al. [33] reported that biofilms are usually resistant to conventional antibiotics; however, an alternative approach to tackling this problem is the use of bacteriocins. This fact has been reported many times, including in our previous studies that tested the susceptibility of biofilm-forming strains or those strains with virulence factor genes to various bacteriocins [34], where most studies reported susceptibility to or inhibition by the bacteriocins used.

The enterococci tested here were mostly susceptible to antibiotics, including susceptibility to vancomycin. This information is promising because the dissemination of VAN resistance could have implications for human health [27]. Enterococci can possess natural (intrinsic) resistance, as in the case of streptomycin, kanamycin and rifampicin resistance in enterococci. In the case of Rif and KAN resistances, they can be chromosomally resistant [35]. Here, the tested strains were mostly gentamicin susceptible; on the other hand, in a U.S. study, for example, Gn resistance was found in 7 out of 18 enterococci isolated from chicken meat [36]. Other antibiotic-resistant enterococci have been reported in meat and dairy products, and even within strains used as a probiotic [37,38].

The studied enterococci were susceptible to gallidermin and nisin, showing higher susceptibility to gallidermin than to nisin. Gallidermin is known to inhibit predominantly Gram-positive strain species, among which enterococci belong [5]. They were also susceptible to durancin, and most strains were resistant to Ent A/P. This means that these enterococci were more susceptible to lantibiotic bacteriocins than to the enterocins used. The resistance of the enterococci tested to Ent A/P could be explained in terms of the way they may be able to produce the same type of antimicrobial substance as Ent A/P (however, neither gene presence nor bacteriocin activity were tested in these strains) [5,6]. Inhibition with ED26E/7 and lantibiotics is, therefore, more highlighted. Using bacteriocins to control/treat biofilm-forming bacteria represents a novel concept in the fight against biofilms [39]. Their prospective use in food production has the potential to lead to the production of safer and healthier foods [12]. In our previous experimental studies, enterocins applied to traditional Slovak bryndza sheep cheese [40] or in Saint-Paulin cheese [41], as well as during the processing of dry fermented Gombasek sausage [42] or Hornad salami that were experimentally [43] infected with *Listeria monocytogenes* and *Listeria innocua,* significantly decreased their count by 2.0 or 4.0 log cycles. Moreover, the present study may also contribute to the food microbiology screening of various marketed products.

## 5. Conclusions

Enterococci from Slovak dry fermented meat products (salami and sausage) were susceptible to treatment with lantibiotic bacteriocins, namely, gallidermin and nisin, where they showed a higher susceptibility to gallidermin than to nisin. They were also susceptible to enterocin–durancin. The results achieved represent a very promising concept in the fight against contaminant, biofilm-forming bacterial strains in the processing of meat products or the products themselves. Moreover, this study also contributes to knowledge of bacteriocin spectra and their potential range of practical applications.

## Figures and Tables

**Table 1:** Enterococci MALDI-TOF identification scores and antibiotic profiles.

Strain	MALDI	Azm	T	Da	Gn	Amp
EF1BS	2.465	R	+20	+20	+15	+11
EF1NS	2.273	R	+17	+14	+15	R
EF2SC	2.271	R	+20	+18	+12	+14
EF2Kal	2.264	R	+20	R	+12	+11
EF4Kal	2.292	R	+10	+16	+10	+10
EFKL5	2.436	R	+14	+20	+11	R
EFPL3	2.095	R	+20	+20	+11	+11
EFPL4	2.247	R	+17	+20	+17	+10
EEPL1S	2.211	R	+15	+14	R	+10
EEKL2	2.307	R	+15	+20	+20	R
EE1Sc	2.401	R	+20	+20	R	+11
EHLov1	2.181	+19	+20	R	+13	+15
EHPL2	2.226	R	R	R	+12	+10
EHTOK1	2.187	R	+20	+11	+16	+15
EHTOK2	2.401	+12	+20	R	+10	R

EF—*E. faecium*; EE—*E. faecalis*, EH—*E. hirae*; MALDI-TOF—matrix–assisted laser desorption ionisation time-of-flight spectrometry identification system; Azm—azithromycin (15 µg), T—tetracycline (30 µg), Da—clindamycin (µg), Gn—gentamicin (120 µg), Amp—ampicillin (10 µg). Strains were susceptible to vancomycin, novobiocin, chloramphenicol, erythromycin and penicillin. They were resistant to kanamycin, rifampicin and streptomycin. R—resistant; +, susceptible; inhibition zone size is given in millimetres.

**Table 2:** Enzyme production in nanomoles (nmoL).

Strains	Alkaline Phosphatase	Esterase (C4)	Esterase Lipase (C8)	Lipase (C14)	Leucin-Arylamidase	Valin-Arylamidase	Cystin-Arylamidase	Trypsin	α-Chymotrypsin	
EF1BS	5	20	10	5	0	0	0	0	0	
EF1NS	5	5	10	5	0	0	0	0	0	
EF2SC	0	20	10	5	10	0	0	0	0	
EF2Kal	0	20	10	5	20	0	0	0	0	
EF4Kal	5	5	5	5	5	0	0	0	0	
EFKL5	5	5	5	0	0	0	0	0	0	
EFPL1s	5	10	10	0	0	0	0	0	0	
EFPL3	5	10	10	5	0	0	0	0	0	
EFPL4	5	10	10	5	0	0	0	0	0	
EEKL2	0	0	0	0	0	0	5	5	5	
EE1Sc	5	20	20	0	5	0	5	0	10	
EHLo1	5	10	20	0	0	0	0	0	0	
EHPL2	0	10	5	5	5	0	0	0	0	
TOK1	0	5	10	0	0	0	0	0	0	
TOK2	5	5	5	0	0	0	0	0	0	
**Strains**	**Acidic Phosphatase**	**Naftol-AS-BI-phospho-hydrolase**	**α-Galactosidase**	**β-Galactosidase**	**β-Glucuronidase**	**α-Glucosidase**	**β-Glucosidase**	***N*-Acetyl-β-glucosamonidase**	**α-Manosidase**	**α-Fucosidase**
EF1BS	5	5	5	5	0	5	5	0	5	5
EF1NS	5	0	5	5	0	5	5	0	5	5
EF2SC	5	0	5	5	0	5	5	0	5	5
EF2Kal	5	0	5	5	0	5	5	0	5	5
EF4Kal	5	0	5	5	0	5	5	0	5	0
EFKL5	0	0	0	5	0	5	5	0	5	5
EFPL1s	10	20	5	5	0	5	5	0	0	0
EFPL3	5	0	5	5	0	5	5	0	5	0
EFPL4	10	0	5	5	5	5	5	0	5	5
EEKL2	5	0	5	5	5	5	5	0	5	5
EE1Sc	10	5	5	5	0	5	5	0	5	5
EHLo1	5	0	5	5	0	5	5	0	5	0
EHPL2	5	0	0	0	0	5	5	0	5	5
TOK1	5	5	5	5	5	5	5	0	5	5
TOK2	5	0	5	5	0	5	5	5	5	5

**Table 3:** Biofilm formation ability and susceptibility to enterocins and lantibiotic bacteriocins expressed in AU/mL.

Strain	Bi	ED	A/P	Gal	Nis
EF1BS	0.117 ± 0.34	3200	ng	6400	1600
EF1NS	0.113 ± 0.30	6400	ng	12,800	3200
EF2SC	0.075 ± 0.03	3200	ng	12,800	3200
EF2Kal	nt	800	ng	25,600	3200
EF4Kal	0.085 ± 0.02	1600	ng	25,600	3200
EFKL5	0.178 ± 0.08	800	ng	3200	3200
EEPL1S	0.115 ± 0.03	6400	100	1600	800
EFPL3	0.217 ± 0.05	3200	100	1600	3200
EFPL4	0.222 ± 0.06	800	ng	1600	3200
EEKL2	0.020 ± 0.00	6400	100	6400	6400
EE1Sc	0.367 ± 0.08	ng	ng	200	3200
EHLov1	0.128 ± 0.04	3200	ng	6400	6400
EHPL2	0.222 ± 0.06	6400	ng	6400	1600
EHTOK1	0.168 ± 0.08	400	100	1600	6400
EHTOK2	0.085 ± 0.06	1600	ng	6400	3200

EF—*E. faecium*; EE—*E. faecalis*, EH—*E. hirae*; Bi—biofilm formation ± SD, ng—negative not inhibited, ED—durancin ED26E/7, A/P—enterocin A/P; gal—gallidermin, Nis—nisin. Inhibition activity against *E. avium* EA 5 was 25,600 AU/mL; nt—not tested.

## Data Availability

All data included in this study are available upon request by contacting the corresponding author.
